# Coalescent Tree Imbalance and a Simple Test for Selective Sweeps Based on Microsatellite Variation

**DOI:** 10.1371/journal.pcbi.1003060

**Published:** 2013-05-16

**Authors:** Haipeng Li, Thomas Wiehe

**Affiliations:** 1 Department of Computational Genomics, CAS Key Laboratory of Computational Biology, CAS-MPG Partner Institute for Computational Biology, Shanghai Institutes for Biological Sciences, Chinese Academy of Sciences, Shanghai, China; 2 Institut für Genetik, Universität zu Köln, Köln, Germany; University of Chicago, United States of America

## Abstract

Selective sweeps are at the core of adaptive evolution. We study how the shape of coalescent trees is affected by recent selective sweeps. To do so we define a coarse-grained measure of tree topology. This measure has appealing analytical properties, its distribution is derived from a uniform, and it is easy to estimate from experimental data. We show how it can be cast into a test for recent selective sweeps using microsatellite markers and present an application to an experimental data set from *Plasmodium falciparum*.

## Author Summary

It is one of the major interests in population genetics to contrast the properties and consequences of neutral and non-neutral modes of evolution. As is well-known, positive Darwinian selection and genetic hitchhiking drastically change the profile of genetic diversity compared to neutral expectations. The present-day observable genetic diversity in a sample of DNA sequences depends on events in their evolutionary history, and in particular on the shape of the underlying genealogical tree. In this paper we study how the shape of coalescent trees is affected by the presence of positively selected mutations. We define a measure of tree topology and study its properties under scenarios of neutrality and positive selection. We show that this measure can reliably be estimated from experimental data, and define an easy-to-compute statistical test of the neutral evolution hypothesis. We apply this test to data from a population of the malaria parasite *Plasmodium falciparum* and confirm the signature of recent positive selection in the vicinity of a drug resistance locus.

## Introduction

The coalescent process is an established tool to describe the evolutionary history of a sample of genes drawn from a natural population [1]–[3]. For a neutrally evolving population of constant size 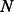 the coalescent has well understood analytical properties concerning tree shape and mutation frequency spectrum which provide a firm basis for a variety of statistical tests of the neutral evolution hypothesis [4]–[8]. Adding recombination as an evolutionary mechanism, the coalescent is usually studied in the framework of the ancestral recombination graph (ARG) [9]. The combined action of selection and recombination has been analyzed first in detail by Hudson and Kaplan [10] and, in terms of genetic hitchhiking, by Kaplan *et al.* [11]. More recently, it was shown that the (non-Markovian) ARG can well be approximated by a simpler, more tractable model, the so-called Sequential Markov Coalescent [12]–[14], which is of particular interest for the efficient simulation of genealogies across large genomic regions. How single recombination events reflect on tree shape under neutrality has recently been analyzed by Ferretti et al. [15]. Here, we concentrate on tree shape in the vicinity of a selected locus.

Selection changes the rate by which coalescent events occur and hence can lead to distortions of tree shape. It is well known [6], [16] that selective sweeps can produce highly unbalanced trees when selection acts in concert with limited recombination, i.e. at some chromosomal distance from the site under selection. Conversely, observing unbalanced trees should provide information about recent selection in a particular genomic region. In fact, this property is also the basis of Li's MDFM test [16]. A practical concern is how such distorted gene genealogies may reliably be estimated or re-constructed using polymorphism data. When working with SNPs a large genomic fragment with many polymorphic sites has to be analyzed to obtain a clear phylogenetic signal. Since for many organisms recombination and mutation rates are on the same order of magnitude [17, Table 4.1], one harvests about as many recombination as polymorphic sites when sampling genomic sequences, thus complicating tree shape estimation. To alleviate this problem one may turn to multi-allelic markers, such as microsatellites, complementing or replacing bi-allelic SNPs.

In this paper we introduce the statistic 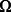 of tree balance and, first, derive theoretical properties of this and derived statistics. Second, we show how a selective sweep affects these statistics. Third, we investigate the possibility and reliability of estimating 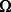 from experimental data. Fourth, we define an easily applicable microsatellite based test statistic for selective sweeps. It requires clustering of microsatellite alleles into two disjoint sets and examining whether these sets are sufficiently different in size and/or whether they have a sufficiently large distance from each other. Finally, we demonstrate a practical application.

### Terminology

**Figure 1:**
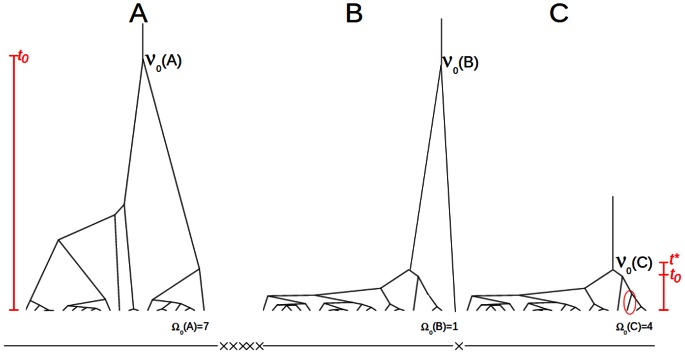
Coalescent trees under recombination and selection. **A**: Sketch of a neutral coalescent tree with tree size 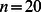. **B** and **C**: A selective sweep in locus C leads to a tree of low height (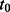 small). The selective sweep was initiated by a beneficial mutation at time 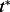. At some distance from C, a single lineage (circled branch in C) has “recombined away” leading to the unbalanced tree shown at locus B. Note that tree height between trees B and C changes drastically and that 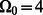 at locus C and 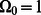 at locus B. Multiple recombination events (indicated by the crosses at the bottom line) between loci A and B lead to essentially uncorrelated trees at A and B.

Consider the coalescent tree for a sample of size 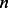. It is a binary tree without left-right orientation, with ordered internal nodes and branch lengths representing a measure of time. All leaves are aligned on the bottom line, representing the present. We use the term *tree topology* when talking about the branching pattern and *tree shape* when talking about topology and branch lengths. We remark that topology and shape can be conceptually distinguished, but in practice estimating topology relies on polymorphism patterns. Since these depend on branch lengths, i.e. on shape, topology can usually not be estimated independently. We call the *size* of a tree the number of leaves and the *length* of a tree the combined length of all branches. The *height* is the time interval between present and root, indicated by 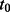 in Figure 1. Let the label of the root be 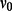. The 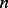 leaves can be grouped into two disjoint sets, 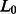 and 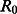, the ‘left-‘ and ‘right-descendants’ of the root. Let 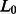 be the smaller of the two sets and 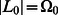. Hence, 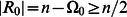. Let 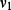 be the ‘right’ child of 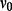, i.e. the root of the subtree with leaf set 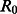. The descendants of 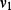 can again be grouped into two disjoint subsets, 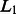 and 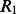, the left- and right-descendants of 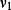. Again, without loss of generality, let 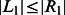 and denote 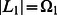. Hence, 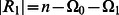. Proceed in this way to define subsets 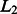, 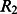, and so on. For any tree there are 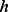 such pairs 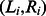 where 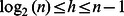, with 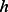 depending on the topology of the tree. The set 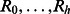 constitutes a – not necessarily unique – top-down sequence of maximal subtrees.

## Results

### Tree topology of the neutral coalescent

Consider a coalescent tree of size 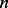 under the neutral model with constant population size, where 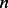 is assumed to be large. Root imbalance is measured by the random variable 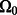. The distribution of 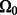 is ‘almost’-uniform [18], [19] on 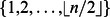. More precisely,
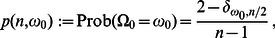
(1)where 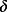.,. denotes here the Kronecker symbol. The expectation is
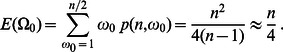
The variance is
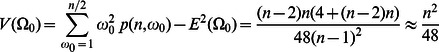
and the standard deviation
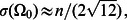
provided 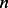 is sufficiently large.

The compound random variables 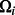, 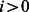, have support which depends on 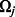, 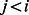. More precisely, the distribution of 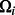, given 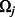, 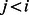, is almost-uniform on 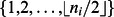 with
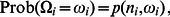
(2)where 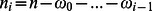 (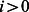) is a random variable which is bounded below by 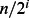 and above by 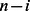. The moments are somewhat more complicated. For instance,
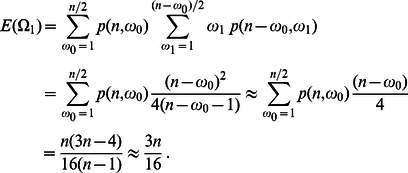
Continuing this way, evaluating sums iteratively and using the above approximation, one derives
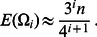
(3)Similary, one can obtain the second moments and combine these to
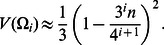
(4)


**Figure 2:**
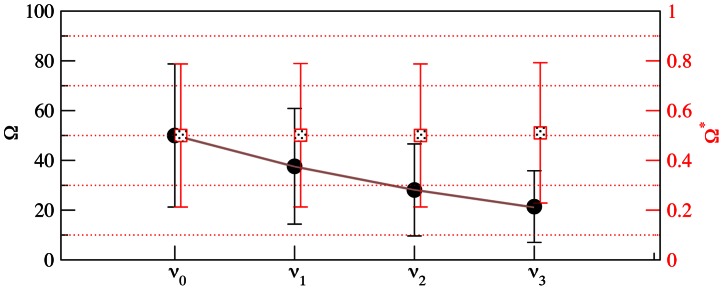
Mean and standard deviation of 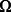 and 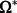 for coalescent trees of size 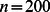. Shown are the values for 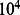 independent realizations. 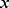-axis: values of 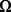 (black circles) and 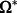 (red squares) are determined for the subtrees originating at node 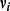, 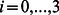. The solid gray line shows the theoretical expectation according to eq (3).

Define now the normalized random variables 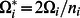. Since 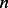 is a constant, we have for 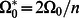


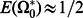
and
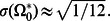
To calculate the moments of 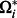, 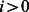, we replace 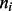 by 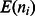. Simulations suggest that this is acceptable, as long as 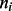 is not too small. Figure 2 shows this fact for 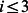. Here we focus on 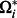 for 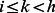, where 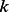 is small and 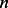 is large (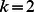, 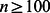, say). Since,
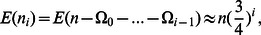
we obtain
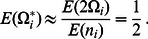
(5)Similarly,
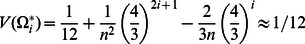
(6)and
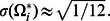
It is very convenient to work with the normalized random variables 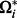 instead of 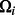. Their support is bounded by 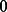 and 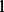 for all 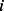 and they are well approximated by independent continuous uniforms on the unit interval. This considerably facilitates the handling of sums and products of 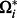. For instance, the joint distribution 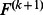 of 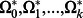 is then approximated by the continuous uniform product with distribution function
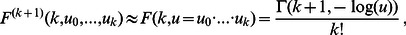
(7)expectation
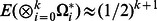
and variance
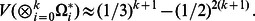
The coefficient of variation, 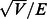, is
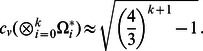
As is well known, the normalized sum of continuous uniforms converges in distribution to a normal random variable rather quickly. In fact, we have for the standardized sum
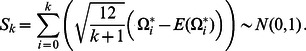
(8)In practice, already 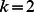 yields a distribution which is reasonably close to a normal (see Suppl. Figure S1).

#### Linked trees

**Figure 3:**
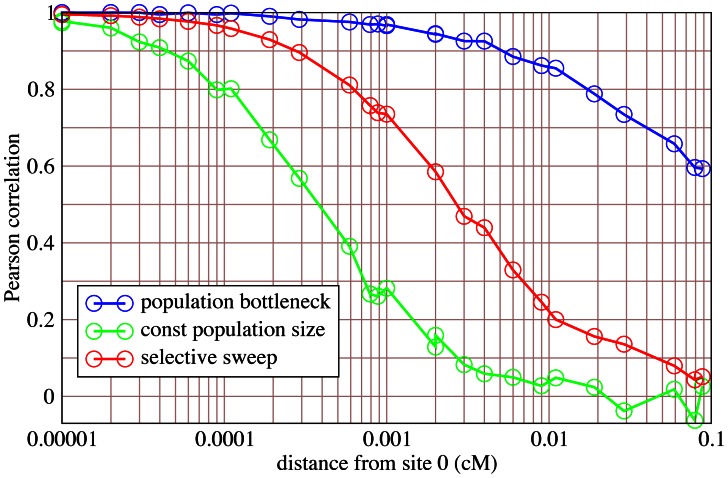
Correlation across distance. Correlation based on simulations (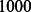 replicates) of the statistic 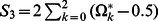 of the true tree. Pearson's correlation coefficient is measured between 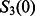 and 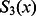 for pairs of trees at position 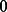 and position 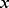. Three scenarios are compared: standard neutral model with constant population size (green), population bottleneck (blue) and selective sweep (red). Sample size 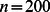, 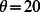, 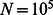 and a recombinaton rate of 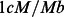 is assumed. The bottleneck parameters are: 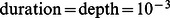, 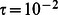. The selective sweep has a strength of 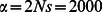. The selected site is at position 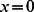. Under standard neutrality, 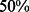 correlation is reached at position 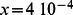 cM, corresponding to about 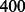 bp.

Consider now a sample of recombining chromosomes. Coalescent trees along a recombining chromosome are not independent. In particular, tree height and tree topology of closely linked trees are highly correlated. However, under conditions of the standard neutral model, correlation breaks down on short distances (Figure 3) [15]. Roughly 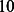 recombination events in the sample history reduce correlation by about 50%. Under neutrality and when 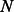 is constant, a sample of size 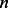 has experienced on average 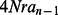 recombination events [20] (Suppl. Figure S2), where 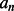 is the 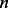-th harmonic number and represents the length of the tree. Assuming a recombination rate of 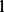 cM/Mb, population size 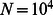 and sample size 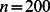, this amounts to roughly 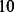 recombination events per 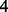 kb. If 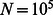, in an interval of only about 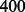 bp correlation is reduced to 50% (Figure 3). Thus, if correlation half-life is determined by roughly 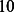 events in the sample, we estimate the correlation length 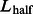 as
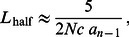
(9)where 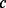 is the recombination rate per 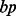 per unit time. Hence, trees may be regarded as essentially uncorrelated when considering physical distances of some 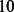 kb and sufficiently large populations and samples.

Eq (9) may be violated if population size 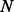 is not constant. As a biologically relevant example we consider a population bottleneck, during which the population is reduced to size 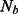. A bottleneck is characterized by three parameters, time of onset, duration (both in units of 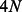) and depth (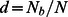). A bottleneck induces time dependent changes of the coalescent rate [21] and a reduction of effective population size. Particularly drastic effects on the genealogy are observed when the duration is similar to or larger than the depth [22]. Given biologically reasonable parameters, this inflation may even be larger under a bottleneck than under a selective sweep (Figure 3).

### Tree topology in the vicinity of a selective sweep

A positively selected allele sweeping through a population leads to a drastic reduction of tree height due to its short fixation time 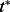 (see Figure 1C). The fixation time depends on the selection coefficient 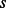 and population size 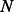. In units of 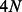, 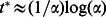, where 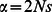 [23]. This is much smaller than the neutral average fixation time 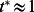. The reduced fixation time leads to a severe reduction of genetic variability. Furthermore, external branches of the tree are elongated relative to internal branches, yielding a star-like phylogeny of an approximate length of 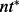. Replacing the neutral tree length 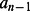 in eq (9) by this figure, we obtain the following estimate for the correlation half-life
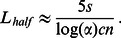
(10)For the parameters used in Figure 3, we have 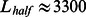 bp, which agrees well with the simulation result.

**Figure 4:**
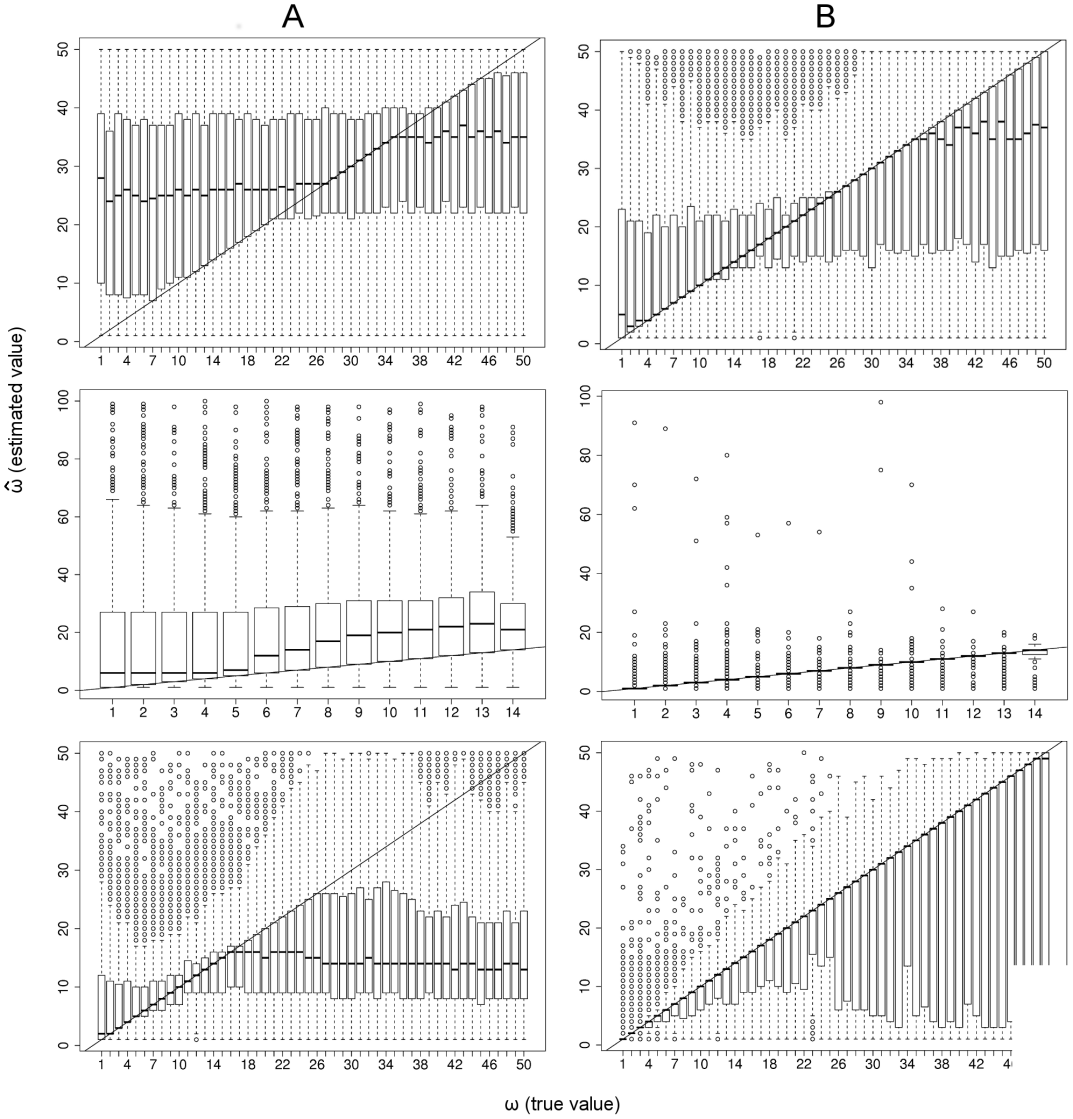
Estimation of 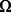. A: estimation of 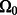 by 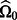. B: estimation of 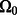 by 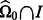. First row: standard neutral model. Second row: Selective sweep; estimation of 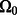 at distance 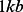 from selected site. Third row: Selective sweep; distance 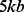 from selected site. Parameters: 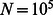; 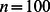 (top and bottom row); 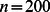 (middle row); 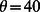; 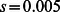; 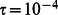.

In contrast to tree height and length, tree topology at the selected site does not necessarily differ from a neutral tree; only when moving away from the sweep site, and with recombination, topology may drastically change. In fact, given a shallow tree, recombination leads with high probability to an increase of tree height and to unbalanced trees [15]. Thus, recombination events next to the selected site tend to increase tree height (see sketch in Figure 1B and C) and to create a bias in favour of unbalanced trees, i.e. trees with small 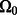 (Figure 4A). The expected *proximal* distance 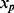 from the selected site of such a recombination event can be estimated as
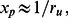
(11)where 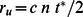, 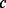 is the per site recombination rate, and 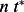 is the length of a star-like phylogeny; the factor 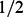 accounts for the fact that it is more likely to recombine with an ancestral chromosome (thereby increasing tree height) as long as these are more abundant than the derived chromosomes carrying the selected allele. Roughly, this is the case during the first half of the fixation time 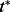. Assuming instead of the star phylogeny a random tree topology of average length 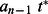 at the selected site, one obtains the larger (call it *distal*) estimate
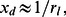
(12)where 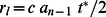.

Unbalanced trees tend to have strongly elongated root branches and harbor an over-abundance of high frequency derived SNP alleles [6], [16]. With microsatellites it is usually not possible to determine the ancestral and derived states of an allele, because they mutate at a high rate and possibly undergo back-mutation. However, under the symmetric single step mutation model, the expected distance between a pair of alleles (in terms of motif copy numbers) behaves as the distance in a one-dimensional symmetric random walk and therefore increases at a rate proportional to the square root of the scaled mutation rate 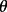 (see Methods). Thus, alleles which are separated by long root branches tend to form two distinct allele clusters.

### Estimating 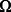


Tree topology is ususally not directly observable and has to be estimated from data. We focus on estimating 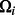, 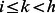, from microsatellite data. Given a sample of 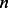 microsatellite alleles with tandem repeat counts 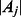, 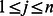, we use UPGMA [24] to construct a hierarchical cluster diagram. If subtree topology within a particular cluster node should not be uniquely re-solvable, for instance if alleles are identical, we randomly assign the alleles of the subtree under consideration to two clusters with equal probability. This gives preference to clusters of balanced size in case of insufficient resolution. We then use the inferred tree topology 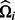 to estimate 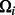 of the true tree. This procedure is conservative for the test statistics described below, since it gives preference to large values 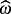 when the true value 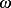 is small (Figure 4, column A). For a cluster pair 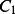, 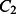, define the distance as
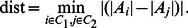
(13)We find that UPGMA clustering gives good estimates of 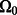 when clusters are clearly separated from each other, i.e. when 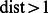. Let 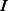 be the indicator variable for this event. Then, we have for the median
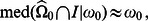
(Figure 4, column B). Without requiring 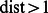 the estimate 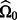 is more biased. In part, this is due to the conservative UPGMA strategy mentioned above. However, estimation of 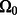 is very accurate when root branches are strongly elongated, i.e. under conditions of selective sweeps or certain bottlenecks (Figure 4, bottom).

### Application: Testing the neutral evolution hypothesis

We now turn to an application of the above results and explain how a new class of microsatellite based tests of the neutral evolution hypothesis can be defined.

Consider a sample of 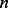 alleles at a microsatellite marker and record their motif repeat numbers. Applying UPGMA clustering to the alleles, we obtain estimates 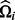, 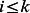 as described above. These are transformed to 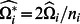. Then, we determine the following test statistics
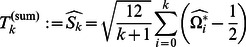
(14)

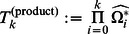
(15)

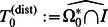
(16)Thus, the test variable 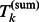 in eq (14) is the estimate of 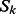 given in eq (8). Similarly, 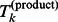 and 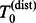 are the estimates of the product 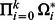 and of 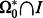.

We now test the null hypothesis 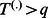 for a critical value 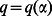. For a given level 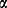 we obtain the critical value 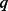 for 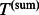 from the standard normal distribution and for 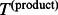 from the uniform product distribution in eq (7) (Table 1). For 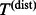 we use the critical value of the normalized version of eq (1). Generally, these critical values are conservative, since 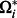 tends to over-estimate 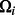, when small (Figure 4). In particular, statistic 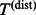 is very conservative due to the additional condition on the distance. The true critical values for level 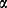 would be larger than those shown in Table 1.

**Table 1:** Critical values for the tests considered in eqs (14)–(16).

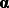	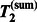	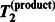	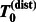
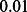	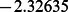	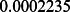	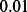
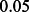	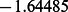	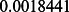	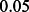

#### False positive rates and power

**Figure 5:**
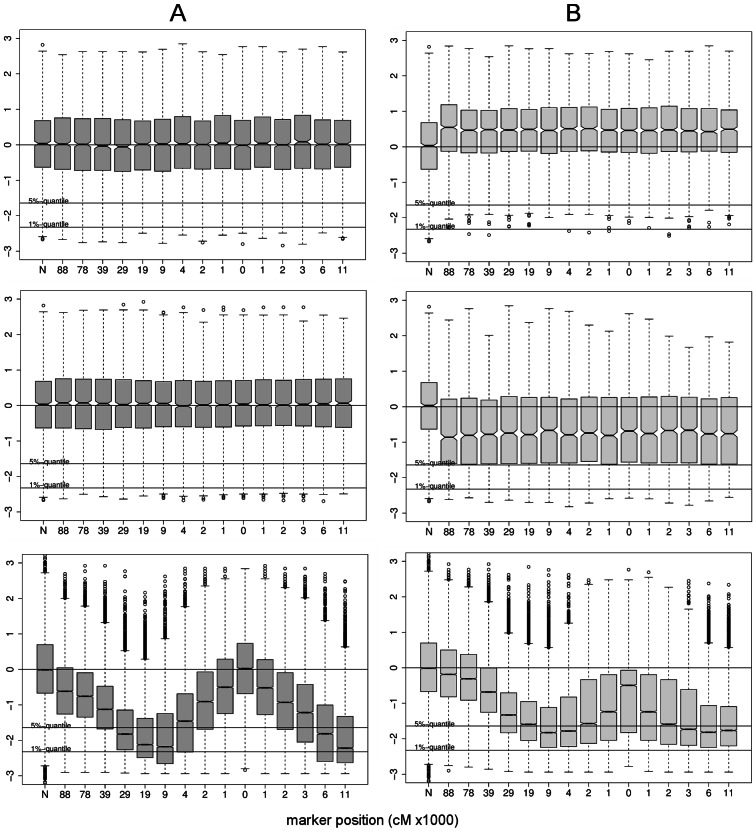
Profile of 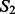 and 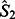 along a recombining chromosome. Plots in column A show the distribution of 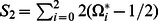, i.e. when the tree topology is known. Plots in column B show the distribution of the estimate 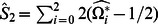 when the tree topology is unknown, but estimated from microsatellite polymorphism data. Each boxplot corresponds to one of 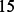 marker loci located at the positions indicated on the 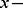
axis. The regions spans 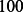 kb in total. Symmetric step-wise mutation model with 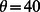. Other parameters: 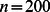, 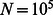 and recombination rate per *bp* 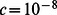 (corresponding to 1 cM/Mb). First row: standard neutral model with constant 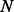. Second row: bottleneck model with severity 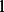 and onset 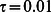. Third row: Selective sweep at locus 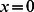 with 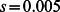 which was completed 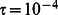 time units ago. For comparison with the theoretical expectation, the leftmost boxplot in each panel shows the standard normal distribution (labeled ‘N’).

**Figure 6:**
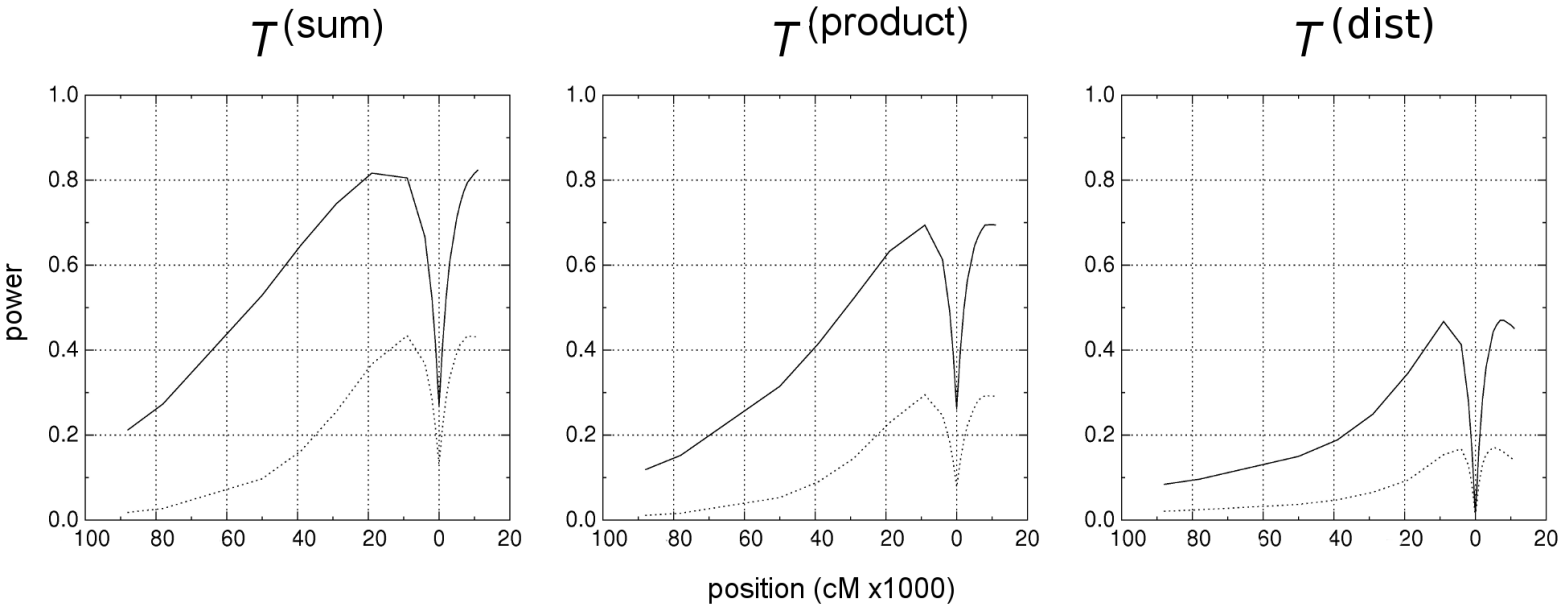
Power to detect loci under recent selection by the three tests defined in eqs (14) to (16) Parameters: level 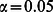 (solid) and .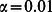 (dotted); selection coefficient 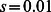; time since fixation 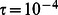; sample size 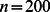; mutation rate 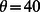; recombination rate 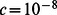. The 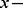
axis shows positions to the left (negative values) and right (positive values) of the locus under selection at position 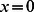. Scale is in cM x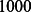, corresponding here to kb.

First, we analyzed the false positive rates under the standard neutral scenario (i.e., constant 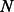) for different mutation rates 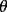 and varying sample sizes 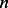. As reference parameter settings for simulations with msmicro (see Methods) we use sample size 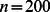, microsatellite mutation rate 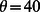 and recombination rate 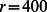. The latter corresponds to a recombination rate of 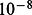 per bp per chromosome, when one assumes a population size of 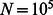 and a size of the investigated genomic region of 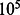 bp (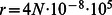). We placed 15 microsatellite markers at positions 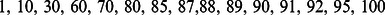 kb. As expected, we find that the false positive rates remain below their theoretical expectation for all parameter choices 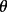 and 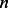 (Figure 5 top; Tables 2 and 3). For the simulations with selection we assumed that a site at position 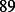 kb was undergoing a selective sweep with selection coefficient 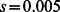 or 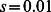. The time since completion of the sweep was an adjustable parameter 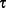, with the reference setting 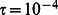. We simulated hard selective sweeps, i.e. the selected allele is introduced as a single copy and fixed with probability about 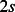. The test statistic 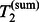 is shown in Figure 5 and power profiles for all three tests in Figure 6. We find that maximum power of the tests is attained within the interval given by eqs (11) and (12) (Figure 6 and Tables 4 and S1). Depending on the strength of selection, maximum power is close to the upper interval bound at 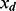 (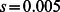, Table S1), or removed from 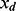 towards the interior of the interval (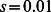, Table 4). This is in agreement with the expectation that only very strong selective sweeps generate a star-like phylogeny, which lead to the proximal estimate 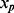 in eq (11). Thus, the location of the power maximum depends on the strength of selection and the details of the tree topology at the selected site. Maximum power for the compound tests 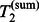 and 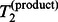 is more removed from the selected site than for the simple test 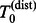. The latter measures imbalance only at the root node 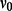 and is most sensitive to single recombination events between marker and selected site, while multiple events blur the effect. The power of all tests is sensitive to the mutation rate and to sample size (Tables S2 and S3). For the parameters tested, the power of the simple 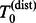 increases when 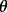 or 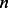 increase. For 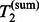, maximum power is reached for 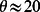. Very small, as well as very high, mutation rates produce little power. Realistic mutation rates in insects and vertebrates are between 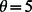 and 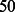 [25]–[27], thus within the powerful domain. Importantly, power can be increased by increasing sample size: all of the above tests become more powerful for large samples (Tables S3, S4 and S5). Since the tests consistently underscore the theoretical false positive rate, relaxed singnificance levels (for instance 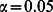) can be applied. At level 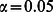 test 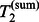 has power of more than 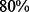 to detect recent selective sweeps (Figure 6 and Table 4). For intermediate mutation rates power of test 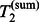 is somewhat higher than of 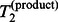 (Table S2). Generally, power profiles of 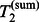 and 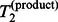 follow qualitatively the same pattern. In contrast, power of test 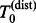 may be quite different. Interestingly, 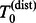 performs better than 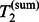 or 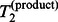 when selection is only moderately strong. Unsurprisingly, power of all tests depends heavily on the strength of selection. Also, the time since completion of the selective sweep influences power. Reasonable power can be reached if 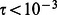 in coalescent units.

**Table 2:** Empirical false positive rate for varying 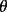.

	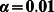			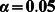		
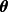	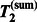	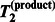	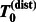	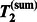	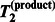	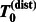
0.1	0.00006	0.00327	0.0001	0.00035	0.01323	0.00047
0.5	0.00523	0.01703	0.00109	0.01724	0.07931	0.00431
1.0	0.01142	0.01749	0.002	0.0463	0.08957	0.00887
1.5	0.01251	0.01414	0.00281	0.06365	0.08425	0.01145
2.0	0.01145	0.01127	0.00355	0.06736	0.07399	0.01354
2.5	0.00933	0.00843	0.00421	0.06579	0.06571	0.01549
3.0	0.00756	0.00663	0.00458	0.06042	0.05718	0.01781
4.0	0.00559	0.00478	0.00534	0.04936	0.04348	0.01884
5.0	0.00415	0.00315	0.0057	0.04073	0.03455	0.0208
10.0	0.00145	0.00131	0.00616	0.0244	0.01889	0.02433
20.0	0.00069	0.00049	0.00632	0.01411	0.01032	0.02685
30.0	0.00064	0.00038	0.00656	0.01018	0.00805	0.02744
**40.0**	0.00043	0.00035	0.00647	0.00839	0.00651	0.02702
50.0	0.00027	0.00031	0.006	0.00828	0.00631	0.02754
100.0	0.00028	0.00033	0.00615	0.00693	0.00591	0.02846
120.0	0.00024	0.00027	0.00614	0.00666	0.00571	0.02806
150.0	0.00024	0.00028	0.00593	0.00699	0.00548	0.02876
200.0	0.00034	0.00026	0.00624	0.00641	0.005	0.02844

Neutral model 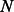 constant, 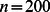, 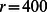. Significance levels 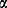 are based on theoretical formulae according to eqs (7) and (8) (reference value indicated in bold).

**Table 3:** Empirical false positive rate for varying sample size 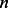.

	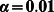			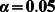		
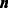	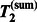	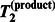	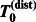	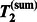	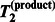	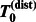
10	N/A	N/A	0.21417	0.0099	N/A	0.21417
20	0.00035	0	0.09527	0.01215	0.00035	0.09527
50	0.00055	0.00003	0.03318	0.0094	0.00286	0.03318
100	0.00052	0.00022	0.0151	0.00925	0.00527	0.02778
150	0.00044	0.00033	0.00902	0.00934	0.00609	0.02411
**200**	0.00039	0.00033	0.00592	0.00943	0.00684	0.02666
300	0.00038	0.00042	0.00388	0.00976	0.00828	0.02282
500	0.00042	0.00055	0.00394	0.01009	0.0093	0.02169
1000	0.00044	0.00104	0.00474	0.01107	0.01148	0.02057

Neutral model 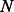 constant, 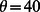, 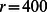. Significance levels 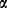 are based on theoretical formulae according to eqs (7) and (8) (reference value indicated in bold).

**Table 4:** Power of 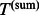, 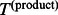 and 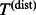 in dependence of distance to selected site.

	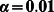			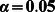			
distance (kb)	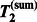	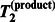	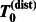	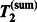	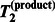	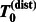	SKD*
−88.0	0.01794	0.01085	0.02051	0.21164	0.11855	0.08392	0.8468
−78.0	0.02708	0.01613	0.02416	0.27325	0.15216	0.09606	0.8873
−50.0	0.09714	0.0528	0.03672	0.52898	0.31465	0.1497	0.9353
−39.0	0.16291	0.09047	0.04749	0.64722	0.41612	0.1887	0.9440
−29.0	0.25581	0.14603	0.06525	0.74461	0.52288	0.24893	0.9435
−19.0	0.3671	0.22901	0.09412	0.81644	0.63249	0.34637	0.9161
−9.0	0.4339	0.29439	0.15377	0.80504	0.69404	0.46718	0.7931
−4.0	0.3679	0.24615	0.16738	0.66659	0.6127	0.41263	0.5969
−2.0	0.28585	0.18531	0.13051	0.52249	0.49368	0.28243	0.4535
−1.0	0.21826	0.1383	0.08574	0.41239	0.39426	0.16926	0.3657
0.0	0.13085	0.07765	0.00971	0.26692	0.26055	0.01182	0.2670
1.0	0.21972	0.13898	0.08428	0.41424	0.39615	0.17043	0.3601
2.0	0.28701	0.18614	0.12943	0.52215	0.49205	0.28118	0.4600
3.0	0.33496	0.22226	0.1548	0.6066	0.56351	0.35927	0.5366
5.0	0.39321	0.26452	0.1706	0.71037	0.64409	0.44278	0.6549
6.0	0.41455	0.28116	0.16901	0.74565	0.66566	0.45932	0.6928
7.0	0.42253	0.28768	0.1645	0.77335	0.68215	0.47001	0.7407
8.0	0.4334	0.29241	0.15955	0.79386	0.69415	0.47023	0.7652
10.0	0.43149	0.29145	0.14693	0.81588	0.69532	0.45901	0.8091
11.0	0.43158	0.2914	0.13982	0.82358	0.69425	0.45046	0.8336

Selective sweep with 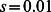, 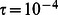, 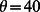, sample size 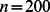.

* SKD-test by Schlötterer *et al.* [37].

We also examined how much the tests are confounded by deviations from the standard neutral model. First, we determined the false positive rates under a population bottleneck. From other studies it is known that bottlenecks with a severity (duration divided by depth) around 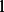 are particularly problematic [16], [28]. We find that tests 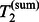 and 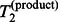 can produce substantially more false positives than expected, in particular if bottlenecks are recent (Table S6). Interestingly, test 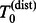 is very robust against these disturbances and the false positive rate remains clearly under the theoretical value for all onset parameters tested (Table S6).

We note that the false positive rates of 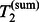 and 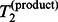 depend strongly on the bottleneck duration even when the severity is kept fixed (Table S7). Very short (duration 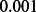), but heavy reductions of 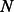 are more disturbing for 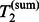 and 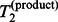 than long, but shallow bottlenecks (duration 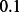). In contrast, 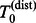 is fairly insensitive to changes of bottleneck duration (Table S7).

Under a model of fast population expansion (expansion rate 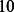), all tests remain below, or close to, their theoretical false positive rate. Again, test 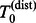 is unsensitive to population expansion and varying onset times (Table S8).

We expected that our topology based tests would yield many false positives under a model of population subdivision. As a potentially critical case we examined sampling from a population divided into two sub-populations which split 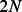 generations ago and which exchange migrants at rate 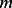. We analyzed both varying migration rates and varying sampling schemes (Tables S9 to S12). The false positive rate for tests 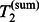 and 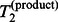 remains clearly under its theoretical expectation, even if sampling is heavily biased (sample size of sub-population 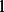 was 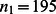 and of sub-population 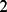 was 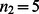; Table S9). In contrast, test 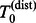, which only measures tree imbalance at the root node, is more vulnerable to biased sampling from a sub-divided population. The false-positive rate grows up to 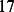
% if 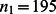 and 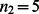. In general, we find test 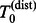 to be less vulnerable to population bottlenecks, but tests 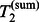 and 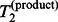 to be more robust under population substructure.

Finally, we examined how deviation from the single step mutation model would influence our tests. We modified the mutation model and allowed occasional jumps (probability 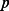) of larger steps. We tested jumps of step size 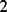 (Table S13) and 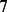 (Table S14). All tests, eminently the compound tests 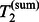 and 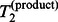, remain clearly below their theoretical false positive rate.

### Case study

**Figure 7:**
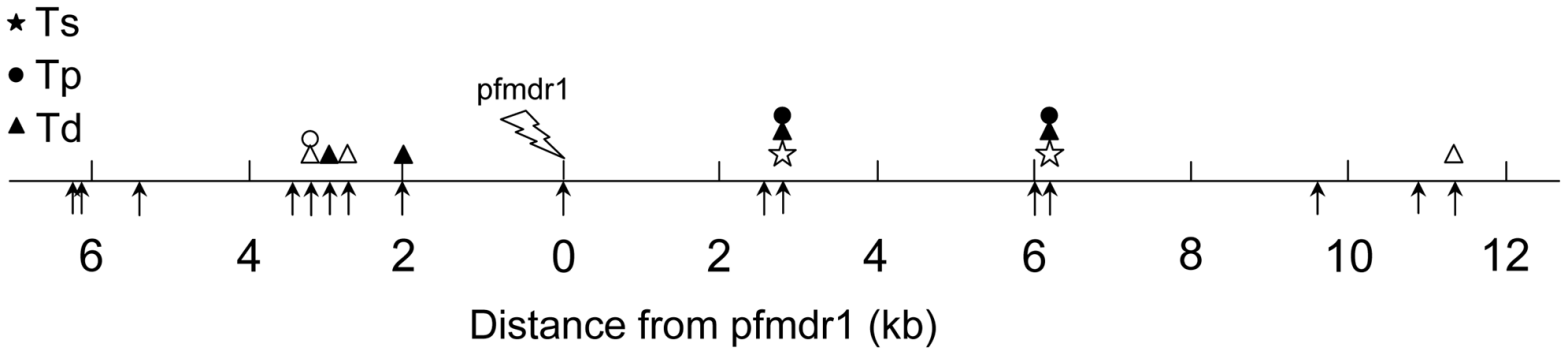
Traces of selection around a drug resistance locus in Plasmodium. Results of tests 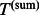 (stars), 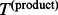 (circles) and 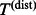 (triangles) applied to a 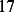 kb region sorrounding the *pfmdr1* locus in *P.falciparum*. Shown are significant results on the 5% (open symbols) and 1% (filled symbols) levels. Positions of the examined microsatellite markers are indicated by arrows. Data from [29].

Emergence of drug resistance in malaria parasites is among the best documented examples for recent selective sweeps. We re-analyzed 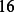 microsatellite markers surrounding a well studied drug resistance locus of malaria parasites [29] (Figure 7). The signature of recent positive selection is consistently detected by all tests on two markers somewhat downstream of the drug resistance locus *pfmdr1* (marker l–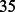 and l–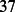 in the notation of [29]; Table 5). Highest significance is reported by test 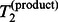 (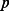-value close to 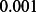). 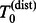 reports a 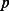-value of 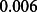 and 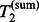 reports 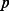-values slightly above 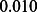. In addition, 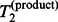 reports locus l–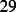 (located upstream of *pfmdr1*) to be significant at 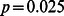. This locus is also detected by 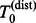 (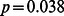). Other four loci are reported only by 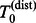 (l–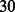 (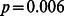), l–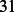 (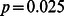), l–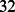 (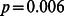), l–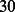 (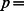) and l–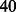 (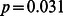)). Discrepancies in the test results are due to their different sensitivities to various parameters. The simple and compound tests have different power profiles with power peaks at different positions from the selected site (Figure 6). Plasmodium in South-East Asia is most likely expanding and sub-structured; however, there is only limited knowledge about the details.

**Table 5:** Test statistics and 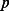-values for the empirical data set of *P.falciparum*.

pos		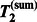	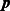 -value	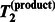	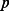 -value	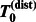		
						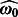	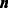	dist^1^
l–25	953,644	−0.1906	0.4244	0.0569	0.4537	146	324	1
l–26	953,768	0.5591	0.7120	0.1235	0.6520	148	322	2
l–27	954,506	−0.7872	0.2156	0.0282	0.3085	95	320	2
l–28	956,456	−1.2289	0.1096	0.0069	0.1268	11	324	3
l–29	956,686	−0.8912	0.1864	0.0007	0.0254 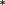	6 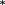	314	4
l–30	956,917	−0.6710	0.2511	0.0019	0.0521	1 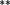	325	3
l–31	957,169	−1.3464	0.0891	0.0030	0.0706	4 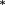	322	4
l–32	957,861	−0.3083	0.3789	0.0024	0.0598	1 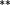	325	31
l–33	**959,894**	0.8260	0.7956	0.0101	0.1629	147	326	2
l–34	962,445	−0.0498	0.4801	0.0611	0.4706	140	326	2
l–35	962,699	−2.1600	0.0154 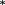	1.9e-5	0.0014 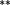	1 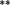	326	23
l–36	965,905	−0.7369	0.2306	0.0337	0.3415	36	326	2
l–37	966,096	−2.2470	0.0123 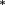	1.4e-5	0.0010 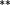	1 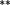	326	9
l–38	969,495	0.3941	0.6533	0.1713	0.7402	117	323	2
l–39	970,775	0.1901	0.5754	0.0528	0.4366	17	322	3
l–40	971,251	−0.8336	0.2023	0.0025	0.0616	5^*^	323	2

Given are the theoretical 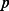-values based on the standard normal (for 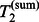) and on the product uniform (for 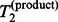) distributions. Values for 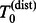 are given as raw data (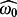, 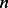, 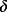). The 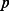-value is 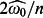. 5% (single star) and 1% (double star) significance are indicated. Marker positions are taken from [29]. The region analyzed (about 17 kb) corresponds to about 1 cM (site under selection in bold).

1 defined in eq (13).

As shown above, 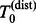 is quite sensitive to biased sampling from different sub-populations. Some of the significant results of 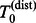 may be inflated due to sub-structure. There is also some disagreement between tests 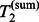 and 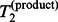 regarding significance, although both test imbalance at tree nodes 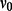, 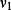 and 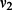. In fact, the cases reported by the two tests may still differ in their details. Comparing the three components 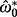, 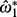 and 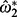 with respect to their maximum and minimum, we find that the cases reported as significant by 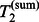 have a 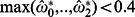 and a 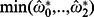 up to 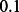. In contrast, for 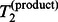, the maximum is close to 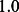 while the minimum tends to be less than 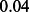 (Figure S4). Thus, test 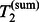 is more restrictive in the sense that all components 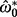, 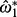 and 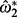 have to be small to yield a significant result. 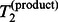 is more permissive and accepts that one of the three components may be large.

All tests agree on significance of two markers close to a site which was previously shown to have experienced a selective sweep. They also agree all on strongly increased 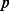-values in the immediate vicinity of the selected site (l–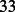, l–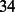). Together, these results confirm the accuracy and practical utility of our tests.

## Discussion

The binary coalescent has a number of well-studied combinatoric and analytic properties [1], [30], [31]. Here we only concentrate on tree topology and use a classic result of Tajima [19] to define a simple measure, 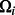, of tree balance. It is the minimum of the left and right subtree sizes under internal node 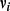. Its normalized version is approximately uniform on the unit interval and the summation over internal nodes 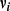, 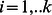, is close to normal. Another summary statistic of tree balance is Colless' index 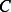 [32]. It also depends on the sizes of left- and right subtrees of the internal nodes, but its distribution is more complicated. 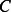 has received attention in the biological literature before [33] and, more recently, in theoretical studies, for instance by Blum&Janson [34]. A problem with Colless' index is that it is difficult to estimate if the true tree structure is unknown. But, limiting attention to the tree structure close to the root, we show that the balance measure 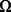 can be estimated, for instance, from microsatellite allele data by a clustering method. We found that a version of UPGMA clustering gives most reliable results.

Coalescent trees for linked loci are not independent. However, correlation dissipates with recombinational distance. In fact, under neutral conditions only about ten recombination events are sufficient to reduce correlation in tree topology by 50%. Thus, estimating tree imbalance at multiple microsatellites can be performed independently for each marker, if they are sufficienty distant from each other. Conversely, with a very small number of recombination events, 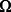 is not drastically altered on average [15]. Thus, when working with SNPs, one may afford to consider haplotype blocks containing a few more recombination events than segragting sites and still be able to reconstruct a reliable gene genealogy. This possibility will be explored in more detail elsewhere.

Microsatellites have been used before as markers for selective sweeps. Schlötterer et al. [35] have proposed the lnRH statistic to detect traces of selection and Wiehe et al. [28] have shown that a multi-locus vesion of lnRH for linked markers can yield high power while keeping false positive rates low. However, a severe practical problem with the lnRH statistic is that it requires data from two populations, and for each of them two additional and independent sets of neutral markers for standardization. There are a few methods to detect deviations from the standard neutral model based on single microsatellite locus data from one population. For instance, the test by Cornuet and Luikart [36], which compares observed and expected heterozygosity, is designed to detect population bottlenecks. A test by Schlötterer et al. [37] uses the number of alleles at a microsatellite locus and determines whether an excess of the number of alleles is due to positive selection (SKD test). However, as the authors pointed out, the test depends critically on a reliable locus-specific estimate of the scaled mutation rate. We have compared SKD and the test proposed here with respect to power and false positive rates. While the SKD-test is generally more powerful, especially at larger distances from the selected site (Table 4 and Suppl. Tables S1, S5), it has higher false positive rates than the tests proposed here, in particular when compared to 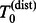 (Suppl. Table S6), and for non-standard mutation models (Suppl. Tables S13, S14). Note also that under population sub-structure SKD yields up to 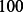 times more false positives than our tests (Suppl. Tables S9 to S12).

It should be emphasized that it is the topology of the underlying genealogical tree, not the genetic variation, which constitutes the basis for the test statistics proposed here. The two steps, estimating topology, and performing the test are two distinct tasks. The quality of the tests hinges on the quality of the re-constructed genealogy. With a perfectly re-constructed genealogy the false positive rates are completely independent from any evolutionary mechanisms which do not affect the average topology, such as historic changes of population size. However, simulations show that power would still remain under 100% in this case. The robustness of topology based tests with respect to demographic changes has been shown before by Li [16] for a similar test which uses SNP data to reconstruct 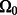. But Li's test can only be performed if an additional non-topological criterion is satisfied and thus can only test a subset of trees with 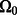. The tests 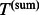 and 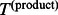 defined here rely only on topological properties of the genelaogy and we argue that multi-allelic markers, such as microsatellites, help estimating the true genealogy and improving test results. Although our analyses and simulations are based on the binary Kingman [1] coalescent, we expect that the new test statistics should be robust also under more general coalescent models, for instance when multiple mergers during the selective sweep phase are allowed [38].

Despite a shift to high throughput sequencing technologies in the last decade, microsatellite typing continues to be a cost-efficient and fast alternative to survey population variability in many experimental studies. This is in particular true for projects directed towards parasite typing, e.g. of Plasmodium, and projects with non-standard model organisms, e.g. social insects [39], [40], but also for many biomedical studies.

## Methods

### Coalescent simulations

We simulated population samples under neutral and hitchhiking models with modified versions of the procedures described by Kim and Stephan [41] and Li and Stephan [42] and of ms [43], termed msmicro. In the modified versions we incorporated evolution of microsatellite loci under the symmetric, single step and multi-step mutation models. Microsatellite mutations are modeled as changes to the number of motif repeats, where only numbers but not particular sequence motifs are recorded. Output data comprise coalescent trees in Newick format and the state of microsatellite alleles for each of 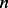 sequences. With msmicro also multiple linked microsatellites can be modeled. Coalescent simulations were run under different evolutionary conditions: neutral with constant population size (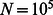), neutral with bottleneck (bottleneck severity 
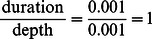
, time since bottleneck 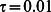), population size expansion (growth rate 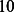), neutral two-island model with migration, and hard selective sweeps (selection 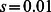 and 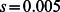, time since fixation of sweep allele 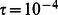).

### Tree topology

Realizations 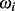 of the ‘true’ random variables 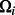, 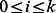 were extracted from the simulation results. Estimation of 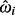 was performed by UPGMA hierarchical clustering. If a cluster node could not be uniquely resolved then we gave preference to a bi-partite partition in which the left and right subtrees were of equal or similar size. This was accomplished by randomly assigning alleles to two clusters with equal probability. To estimate 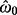 we also explored a simple clustering method which works in the following way: we first sorted alleles by size; then we divided the sorted list into two halfs. The separator was placed between those two alleles which had maximal distance (in terms of microsatellite repeat units) from each other. If this was not unique, the separator was placed between those two alleles that resulted in two sets of most similar size. While this clustering method is very effective in estimating 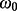, it is less accurate than UPGMA clustering for 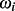, 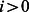.

### Distance between microsatellite alleles

The single step symmetric mutation model behaves as a one-dimensional symmetric random walk of step size one. The theory of random walks (e.g. [44]) tells that the average distance between the origin of the walk and the current position scales with the square root of the number 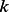 of steps. More precisely,
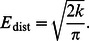
The variance is linear in 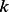. Here, steps are represented by mutational events occuring at rate 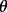. Thus, 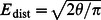 and 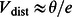, where 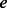 is Euler's constant. The empirical distance between two clusters 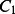 and 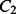 can be calculated as
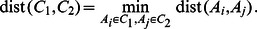



## Supporting Information

Figure S1
**Agreement of** 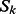 **with the standard normal.** Shown are the distribution functions for the standard normal distribution (green line), and for (see eq (8)) 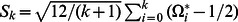, 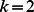 (red line) and 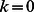 (blue line). The latter is uniform on 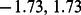. Obviously, already for 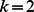 the agreement between the standard normal and 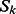 is quite good.(EPS)

Figure S2
**Average number of recombination events in neutral coalescent trees.** (A) in dependence of sample size 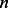 (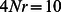) and (B) of the scaled recombination rate 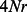 (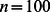). Red: simulation results obtained from 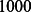 replicates of ms [43]. Shown are average (bullets) and standard deviation (whiskers). Black: theoretical value 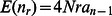.(EPS)

Figure S3
**Distance from sweep site to first recombination site.** Given that the rate of the first recombination event adjacent to a selective sweep site is 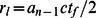 (in case of a neutral topology) or 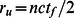 (in case of a star phylogeny) the distance between the selected site and the ‘first’ recombination event is described by a Poisson process with rate 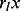 or 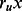. Shown is the probability that the Poissson variable is 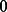 (i.e., for a “recombination free zone”) for 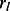 (upper curve) and 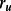 (lower curve).(EPS)

Figure S4
**Differences between tests (A)** 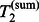 **and (B)** 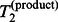 **.** Given a test is significant at level 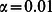, the plots show the maximum (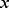-axis) and the minimum (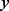-axis) of the three terms 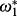, 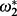 and 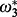, which enter into the sum and product in 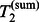 and 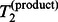, respectively. The sum- and product-tests may yield different results, because the summands are differently constrained (here (A), the maximum 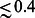) than the factors (here (B), the maximum may reach almost 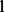, but the minimum is smaller than in the sum-test).(PDF)

Table S1
**Power of** 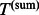 **,** 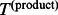 **and** 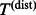 **in dependence of distance to selected site.** Moderate selection strength.(PDF)

Table S2
**Power of** 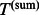 **,** 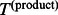 **and** 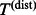 **in dependence of mutation rate** 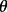 **.**
(PDF)

Table S3
**Power of** 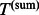 **,** 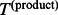 **and** 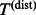 **in dependence of sample size** 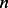 **.**
(PDF)

Table S4
**Power of** 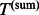 **,** 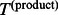 **and** 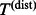 **in dependence of distance to selected site.** Small sample size.(PDF)

Table S5
**Power of** 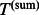 **,** 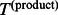 **and** 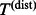 **in dependence of distance to selected site.** Large sample size.(PDF)

Table S6
**Empirical false positive rate. Bottleneck model** with varying onset 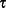 of the bottleneck. Strength is fixed at 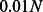. Significance levels 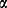 are based on theoretical formulae according to eqs (7) and (8).(PDF)

Table S7
**Empirical false positive rate. Bottleneck model** with varying duration of the bottleneck. Severity (duration divided by strength) is fixed at 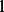. Significance levels 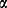 are based on theoretical formulae according to eqs (7) and (8).(PDF)

Table S8
**Empirical false positive rate. Population expansion** with varying onset 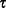 of the expansion. Expansion rate is fixed at 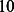.(PDF)

Table S9
**Empirical false positive rate. Population sub-structure** with two sub-populations, split time 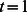 in the past and sampling scheme 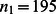, 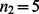. Varying migration rate 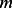 per generation per 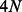 individuals. Significance levels 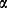 are based on theoretical formulae according to eqs (7) and (8).(PDF)

Table S10
**Empirical false positive rate. Population sub-structure** with two sub-populations, split time 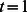 in the past and sampling scheme 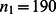, 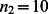. Varying migration rate 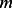 per generation per 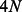 individuals. Significance levels 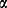 are based on theoretical formulae according to eqs (7) and (8).(PDF)

Table S11
**Empirical false positive rate. Population sub-structure** with two sub-populations, split time 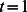 in the past and sampling scheme 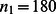, 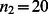. Varying migration rate 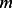 per generation per 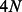 individuals. Significance levels 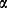 are based on theoretical formulae according to eqs (7) and (8).(PDF)

Table S12
**Empirical false positive rate. Population sub-structure** with two sub-populations, split time 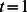 in the past and sampling scheme 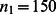, 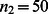. Varying migration rate 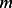 per generation per 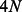 individuals. Significance levels 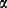 are based on theoretical formulae according to eqs (7) and (8).(PDF)

Table S13
**Empirical false positive rate. Mutation model with jumps of size** 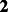. Varying probability 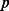 for a step of size 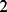. With probability 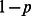 the step size is 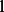. Significance levels 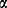 are based on theoretical formulae according to eqs (7) and (8).(PDF)

Table S14
**Empirical false positive rate. Mutation model with jumps of size** 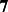. Varying probability 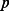 for a step of size 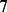. With probability 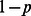 the step size is 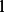. Significance levels 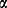 are based on theoretical formulae according to eqs (7) and (8).(PDF)
