# Supplementary figures and images for: Coalescent Tree Imbalance and a Simple Test for Selective Sweeps Based on Microsatellite Variation
Source: PLoS Comput Biol. 2013 May 16;9(5):e1003060. doi: 10.1371/journal.pcbi.1003060 (PMC3656098; doi:10.1371/journal.pcbi.1003060)

**A**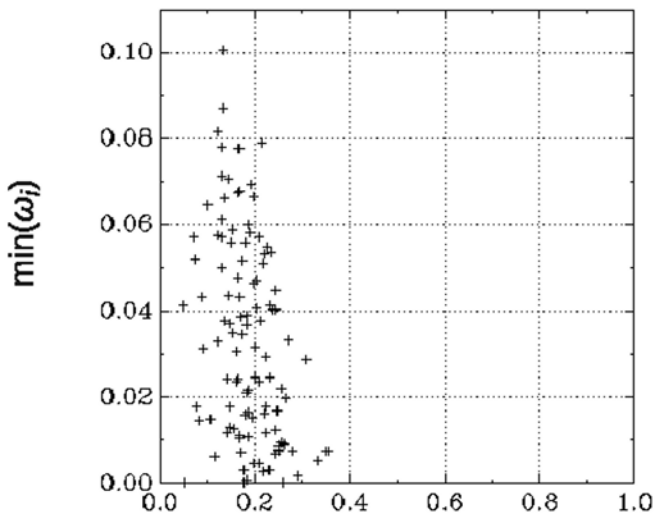**B**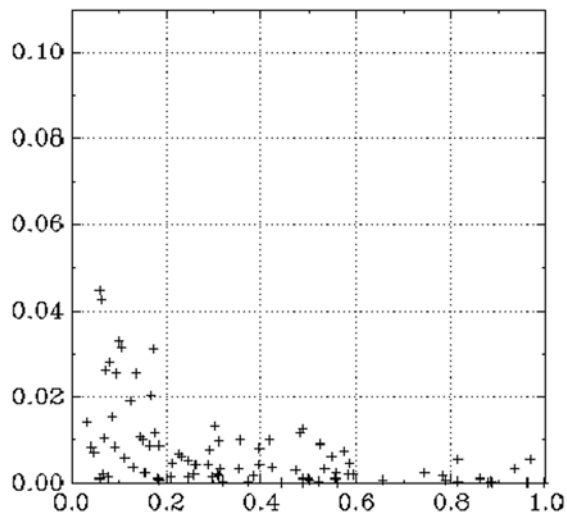

Supplement: Figure S4 — Differences between tests (A) and (B) . Given a test is significant at level , the plots show the maximum (-axis) and the minimum (-axis) of the three terms , and , which enter into the sum and product in and , respectively. The sum- and product-tests may yield different results, because the summands are differently constrained (here (A), the maximum ) than the factors (here (B), the maximum may reach almost , but the minimum is smaller than in the sum-test). (PDF) [file pcbi.1003060.s004.pdf]
