# Supplementary material for: Coalescent Tree Imbalance and a Simple Test for Selective Sweeps Based on Microsatellite Variation
Source: PLoS Comput Biol. 2013 May 16;9(5):e1003060. doi: 10.1371/journal.pcbi.1003060 (PMC3656098; doi:10.1371/journal.pcbi.1003060)
Supplement: Table S1 — Power of , and in dependence of distance to selected site. Moderate selection strength. (PDF) [file pcbi.1003060.s005.pdf]

Table S1. Power of $T^{(\mathbf{sum})}$, $T^{(\mathbf{product})}$ and $T^{(\mathbf{dist})}$ in dependence of distance to selected site. Moderate selection strength.

	$\alpha = 0.01$			$\alpha = 0.05$			
distance (kb)	$T_2^{(\text{sum})}$	$T_2^{(\text{product})}$	$T_0^{(\text{dist})}$	$T_2^{(\text{sum})}$	$T_2^{(\text{product})}$	$T_0^{(\text{dist})}$	SKD*
-88.0	0.0024	0.00198	0.01205	0.03773	0.02547	0.04918	0.38978
-78.0	0.00352	0.00249	0.01219	0.05101	0.03337	0.05231	0.46468
-50.0	0.01279	0.0082	0.01861	0.14195	0.08394	0.08038	0.70152
-39.0	0.02603	0.01576	0.02434	0.22844	0.13637	0.10191	0.77336
-29.0	0.04959	0.03123	0.03224	0.33846	0.20899	0.13661	0.81974
-19.0	0.09292	0.06603	0.04913	0.47323	0.32176	0.19855	0.82495
-9.0	0.1528	0.12917	0.0875	0.58321	0.44846	0.31711	0.69911
-4.0	0.14253	0.13536	0.11036	0.55597	0.43924	0.3397	0.47106
-2.0	0.10248	0.10226	0.09572	0.47656	0.3768	0.25917	0.30610
-1.0	0.06848	0.06854	0.06862	0.41117	0.31957	0.16987	0.20460
0.0	0.01823	0.01552	0.01383	0.3088	0.22456	0.01858	0.09020
1.0	0.07008	0.07046	0.0699	0.41566	0.32085	0.17263	0.20603
2.0	0.10457	0.1033	0.09726	0.47807	0.38001	0.2611	0.30748
3.0	0.12845	0.1248	0.107	0.52423	0.41668	0.31387	0.39475
5.0	0.15041	0.14094	0.10696	0.57279	0.45309	0.34652	0.53361
6.0	0.15732	0.14268	0.10273	0.58033	0.45513	0.34908	0.58359
7.0	0.15761	0.14028	0.0998	0.58929	0.45991	0.34365	0.63050
8.0	0.15605	0.13543	0.09382	0.5908	0.45563	0.33244	0.67039
10.0	0.14787	0.12186	0.08277	0.57952	0.43868	0.30483	0.72507
11.0	0.14231	0.11471	0.07802	0.56758	0.42758	0.28931	0.74858

Selective sweep with $s = 0.005$, $\tau = 10^{-4}$, $\theta = 40$, sample size $n = 200$.

* SKD-test by Schlötterer *et al.* [37]
