# Supplementary material for: Coalescent Tree Imbalance and a Simple Test for Selective Sweeps Based on Microsatellite Variation
Source: PLoS Comput Biol. 2013 May 16;9(5):e1003060. doi: 10.1371/journal.pcbi.1003060 (PMC3656098; doi:10.1371/journal.pcbi.1003060)
Supplement: Table S2 — Power of , and in dependence of mutation rate . (PDF) [file pcbi.1003060.s006.pdf]

Table S2. Power of $T^{(\mathbf{sum})}$, $T^{(\mathbf{product})}$ and $T^{(\mathbf{dist})}$ in dependence of mutation rate θ.

	$\alpha = 0.01$			$\alpha = 0.05$		
θ	$T_2^{(sum)}$	$T_2^{(product)}$	$T_0^{(dist)}$	$T_2^{(sum)}$	$T_2^{(product)}$	$T_0^{(dist)}$
0.1	0.00002	0.00217	0.00083	0.00002	0.00535	0.00226
0.5	0.00179	0.03451	0.00831	0.00187	0.08217	0.02356
1.0	0.01059	0.08174	0.01697	0.01117	0.2093	0.04672
1.5	0.02633	0.11888	0.02419	0.02748	0.32257	0.06492
2.0	0.04879	0.14453	0.02802	0.0511	0.4109	0.07628
2.5	0.0717	0.16441	0.03246	0.07546	0.47518	0.08988
3.0	0.09814	0.17805	0.03528	0.10385	0.5203	0.09767
4.0	0.14893	0.19934	0.04269	0.15772	0.558	0.11533
5.0	0.18859	0.20936	0.04537	0.20354	0.55163	0.12759
10.0	0.30562	0.22845	0.06514	0.35658	0.43331	0.18098
20.0	0.26084	0.15494	0.08454	0.49867	0.44754	0.22933
30.0	0.14708	0.11109	0.09119	0.53301	0.43122	0.24783
40.0	0.10262	0.1027	0.09591	0.47789	0.37554	0.26007
50.0	0.07607	0.0942	0.09868	0.382	0.32858	0.2699
100.0	0.03918	0.05419	0.10842	0.24881	0.25069	0.29666
120.0	0.03813	0.04841	0.11072	0.20164	0.23363	0.30353
150.0	0.03289	0.0416	0.11154	0.16112	0.21642	0.30764
200.0	0.02797	0.03672	0.11381	0.14909	0.19115	0.31096

Selective sweep with $s = 0.005$, $\tau = 10^{-4}$, $n = 200$,
distance to selected position $x = 10kb$.
