# Supplementary material for: Coalescent Tree Imbalance and a Simple Test for Selective Sweeps Based on Microsatellite Variation
Source: PLoS Comput Biol. 2013 May 16;9(5):e1003060. doi: 10.1371/journal.pcbi.1003060 (PMC3656098; doi:10.1371/journal.pcbi.1003060)
Supplement: Table S3 — Power of , and in dependence of sample size . (PDF) [file pcbi.1003060.s007.pdf]

Table S3. Power of $T^{(\mathbf{sum})}$, $T^{(\mathbf{product})}$ and $T^{(\mathbf{dist})}$ in dependence of sample size n.

	$\alpha = 0.01$			$\alpha = 0.05$		
n	$T_2^{(\text{sum})}$	$T_2^{(\text{product})}$	$T_0^{(\text{dist})}$	$T_2^{(\text{sum})}$	$T_2^{(\text{product})}$	$T_0^{(\text{dist})}$
10	N/A	N/A	0.13996	0.02244	N/A	0.13996
20	0.00922	0	0.14474	0.11729	0.00922	0.14474
50	0.04367	0.00589	0.14589	0.27807	0.12065	0.14589
100	0.06891	0.04228	0.13104	0.38301	0.25216	0.19846
150	0.08789	0.07622	0.11057	0.44456	0.33223	0.22271
200	0.10365	0.10255	0.0953	0.47803	0.37523	0.25947
300	0.12471	0.13835	0.07524	0.51983	0.43151	0.26828
500	0.15007	0.18279	0.09271	0.56083	0.48136	0.28552
1000	0.17424	0.23261	0.11754	0.59588	0.52515	0.296

Selective sweep with $s = 0.005$, $\tau = 10^{-4}$, $\theta = 40$,
distance to selected position $x = 10kb$.
N/A: test cannot be computed due to small sample size
