# Supplementary material for: Coalescent Tree Imbalance and a Simple Test for Selective Sweeps Based on Microsatellite Variation
Source: PLoS Comput Biol. 2013 May 16;9(5):e1003060. doi: 10.1371/journal.pcbi.1003060 (PMC3656098; doi:10.1371/journal.pcbi.1003060)
Supplement: Table S4 — Power of , and in dependence of distance to selected site. Small sample size. (PDF) [file pcbi.1003060.s008.pdf]

Table S4. Power of $T^{(\mathbf{sum})}$, $T^{(\mathbf{product})}$ and $T^{(\mathbf{dist})}$ in dependence of distance to selected site. Small sample size.

	$\alpha = 0.01$			$\alpha = 0.05$		
distance (kb)	$T_2^{(\mathrm{sum})}$	$T_2^{(\mathrm{product})}$	$T_0^{(\mathrm{dist})}$	$T_2^{(\mathrm{sum})}$	$T_2^{(\mathrm{product})}$	$T_0^{(\mathrm{dist})}$
-88.0	0.00269	0.00025	0.05016	0.0388	0.01095	0.05016
-78.0	0.0034	0.00042	0.05501	0.04928	0.01456	0.05501
-50.0	0.01252	0.00129	0.07919	0.13078	0.04168	0.07919
-39.0	0.02307	0.00264	0.09408	0.19491	0.06638	0.09408
-29.0	0.04071	0.00571	0.12204	0.2792	0.1048	0.12204
-19.0	0.06758	0.01005	0.16465	0.377	0.15966	0.16465
-9.0	0.08159	0.01407	0.22299	0.4181	0.19573	0.22299
-4.0	0.06312	0.00988	0.20199	0.35016	0.16047	0.20199
-2.0	0.0439	0.00606	0.14783	0.27544	0.11975	0.14783
-1.0	0.03031	0.00394	0.09747	0.22061	0.08958	0.09747
0.0	0.01373	0.0009	0.02524	0.15324	0.05191	0.02524
1.0	0.03074	0.00348	0.09601	0.22469	0.09033	0.09601
2.0	0.04391	0.00585	0.14744	0.27455	0.11968	0.14744
3.0	0.05505	0.00807	0.18097	0.31678	0.14332	0.18097
5.0	0.07063	0.01153	0.21375	0.37395	0.17395	0.21375
6.0	0.07691	0.0125	0.22455	0.3912	0.18487	0.22455
7.0	0.07943	0.0135	0.22612	0.40368	0.19123	0.22612
8.0	0.08106	0.014	0.22627	0.41355	0.19558	0.22627
10.0	0.08232	0.01407	0.21908	0.42148	0.19508	0.21908
11.0	0.08491	0.01438	0.21251	0.4211	0.19677	0.21251

Selective sweep with $s = 0.005$, $\tau = 10^{-4}$, $\theta = 40$, sample size $n = 50$.
