# Supplementary material for: Coalescent Tree Imbalance and a Simple Test for Selective Sweeps Based on Microsatellite Variation
Source: PLoS Comput Biol. 2013 May 16;9(5):e1003060. doi: 10.1371/journal.pcbi.1003060 (PMC3656098; doi:10.1371/journal.pcbi.1003060)
Supplement: Table S5 — Power of , and in dependence of distance to selected site. Large sample size. (PDF) [file pcbi.1003060.s009.pdf]

Table S5. Power of $T^{(\mathbf{sum})}$, $T^{(\mathbf{product})}$ and $T^{(\mathbf{dist})}$ in dependence of distance to selected site. Large sample size.

	$\alpha = 0.01$			$\alpha = 0.05$			
distance (kb)	$T_2^{(\text{sum})}$	$T_2^{(\text{product})}$	$T_0^{(\text{dist})}$	$T_2^{(\text{sum})}$	$T_2^{(\text{product})}$	$T_0^{(\text{dist})}$	SKD*
-88.0	0.00247	0.00353	0.00764	0.04152	0.03311	0.03966	0.3931
-78.0	0.00348	0.00462	0.00863	0.05346	0.04216	0.0435	0.4833
-50.0	0.01398	0.01366	0.01386	0.15285	0.10515	0.06729	0.7059
-39.0	0.02658	0.0246	0.0181	0.23877	0.15918	0.08554	0.7829
-29.0	0.05434	0.0491	0.02617	0.36086	0.24444	0.11945	0.8414
-19.0	0.10551	0.0992	0.03976	0.50899	0.3739	0.17977	0.8532
-9.0	0.18414	0.19212	0.07298	0.63174	0.52356	0.30889	0.7594
-4.0	0.19177	0.22401	0.10218	0.62366	0.53774	0.35723	0.5294
-2.0	0.14858	0.18127	0.09373	0.5579	0.4801	0.2819	0.3253
-1.0	0.10237	0.13074	0.06664	0.49192	0.41612	0.18398	0.2103
0.0	0.03133	0.04752	0.00709	0.38575	0.3077	0.00912	0.0582
1.0	0.10293	0.13245	0.06732	0.49034	0.41642	0.1875	0.2012
2.0	0.14956	0.18541	0.09428	0.56146	0.48276	0.28691	0.3310
3.0	0.17586	0.20905	0.10077	0.6022	0.51759	0.33372	0.4418
5.0	0.19906	0.227	0.09861	0.63721	0.54612	0.36092	0.5969
6.0	0.19993	0.22093	0.09322	0.64041	0.54491	0.35334	0.6553
7.0	0.19492	0.21297	0.08549	0.64123	0.5414	0.33872	0.6930
8.0	0.18806	0.20402	0.0795	0.6384	0.53484	0.32225	0.7345
10.0	0.17526	0.18338	0.06743	0.62424	0.51089	0.29035	0.7844
11.0	0.1668	0.17187	0.06374	0.61307	0.49752	0.27407	0.8028

Selective sweep with $s = 0.005$, $\tau = 10^{-4}$, $\theta = 40$, sample size $n = 500$.

* SKD-test by Schlötterer *et al.* [37]
