# Supplementary material for: Coalescent Tree Imbalance and a Simple Test for Selective Sweeps Based on Microsatellite Variation
Source: PLoS Comput Biol. 2013 May 16;9(5):e1003060. doi: 10.1371/journal.pcbi.1003060 (PMC3656098; doi:10.1371/journal.pcbi.1003060)
Supplement: Table S6 — Empirical false positive rate. Bottleneck model with varying onset of the bottleneck. Strength is fixed at . Significance levels are based on theoretical formulae according to eqs (7) and (8). (PDF) [file pcbi.1003060.s010.pdf]

Table S6. Empirical false positive rate. Bottleneck model with varying onset τ of the bottleneck. Strength is fixed at $0.01N$. Significance levels α are based on theoretical formulae according to eqs (7) and (8).

	$\alpha = 0.01$			$\alpha = 0.05$			
τ	$T_2^{(sum)}$	$T_2^{(product)}$	$T_0^{(dist)}$	$T_2^{(sum)}$	$T_2^{(product)}$	$T_0^{(dist)}$	SKD*
$1\ 10^{-4}$	0.02282	0.03524	0.00655	0.07318	0.12431	0.03148	0.10711
$2\ 10^{-4}$	0.03035	0.05088	0.00641	0.09171	0.16072	0.03078	0.12756
$5\ 10^{-4}$	0.04846	0.08163	0.0062	0.13872	0.24866	0.0299	0.17234
0.0010	0.06563	0.09262	0.00655	0.18736	0.32287	0.02934	0.21512
0.0020	0.07371	0.06852	0.00651	0.22373	0.31886	0.02813	0.25856
0.0050	0.0622	0.03664	0.00758	0.22119	0.18918	0.02802	0.25851
0.01	0.02998	0.0161	0.00896	0.19415	0.14563	0.02849	0.24116
0.02	0.01045	0.0094	0.00962	0.11888	0.08584	0.02759	0.18383
0.05	0.00308	0.00252	0.0092	0.04763	0.03502	0.02734	0.07976
0.1	0.00104	0.00098	0.00792	0.02201	0.01651	0.02593	0.04048
0.2	0.0006	0.00052	0.00713	0.01193	0.00972	0.02441	0.02766
0.5	0.00041	0.0003	0.00554	0.00849	0.00616	0.02259	0.0334
1.0	0.00024	0.00031	0.00586	0.00852	0.00631	0.02481	0.04479
100.0	0.0003	0.00031	0.00629	0.00901	0.00698	0.02782	0.05377

* SKD-test from [37].
