# Supplementary material for: Coalescent Tree Imbalance and a Simple Test for Selective Sweeps Based on Microsatellite Variation
Source: PLoS Comput Biol. 2013 May 16;9(5):e1003060. doi: 10.1371/journal.pcbi.1003060 (PMC3656098; doi:10.1371/journal.pcbi.1003060)
Supplement: Table S7 — Empirical false positive rate. Bottleneck model with varying duration of the bottleneck. Severity (duration divided by strength) is fixed at . Significance levels are based on theoretical formulae according to eqs (7) and (8). (PDF) [file pcbi.1003060.s011.pdf]

Table S7. Empirical false positive rate. Bottleneck model with varying duration of the bottleneck. Severity (duration divided by strength) is fixed at 1. Significance levels α are based on theoretical formulae according to eqs (7) and (8).

	$\alpha = 0.01$			$\alpha = 0.05$		
duration	$T_2^{(\mathrm{sum})}$	$T_2^{(\mathrm{product})}$	$T_0^{(\mathrm{dist})}$	$T_2^{(\mathrm{sum})}$	$T_2^{(\mathrm{product})}$	$T_0^{(\mathrm{dist})}$
0.0010	0.04559	0.02314	0.00934	0.23533	0.17179	0.02909
0.01	0.02976	0.01627	0.00934	0.19411	0.14517	0.029
0.1	0.00468	0.00325	0.00667	0.0532	0.04092	0.0242
