# Supplementary material for: Coalescent Tree Imbalance and a Simple Test for Selective Sweeps Based on Microsatellite Variation
Source: PLoS Comput Biol. 2013 May 16;9(5):e1003060. doi: 10.1371/journal.pcbi.1003060 (PMC3656098; doi:10.1371/journal.pcbi.1003060)
Supplement: Table S8 — Empirical false positive rate. Population expansion with varying onset of the expansion. Expansion rate is fixed at . (PDF) [file pcbi.1003060.s012.pdf]

Table S8. Empirical false positive rate. Population expansion with varying onset τ of the expansion. Expansion rate is fixed at 10.

	$\alpha = 0.01$			$\alpha = 0.05$			
t	$T_2^{(\text{sum})}$	$T_2^{(\text{product})}$	$T_0^{(\text{dist})}$	$T_2^{(\text{sum})}$	$T_2^{(\text{product})}$	$T_0^{(\text{dist})}$	SKD*
$1\ 10^{-4}$	0.00593	0.00464	0.00495	0.04994	0.04341	0.01912	0.0540
$2\ 10^{-4}$	0.00538	0.00439	0.00462	0.0498	0.04366	0.01785	0.0571
$5\ 10^{-4}$	0.00553	0.0043	0.00474	0.04914	0.04511	0.01705	0.0538
0.0010	0.00546	0.0046	0.00487	0.05308	0.04848	0.01625	0.0609
0.0020	0.00526	0.00382	0.0052	0.05226	0.04673	0.01545	0.0510
0.0050	0.00483	0.00366	0.00595	0.05112	0.04475	0.01429	0.0454
0.01	0.00401	0.003	0.00624	0.04519	0.03834	0.01406	0.0393
0.02	0.00202	0.00182	0.00738	0.03759	0.02988	0.0156	0.0265
0.05	0.00101	0.00106	0.00781	0.02135	0.01816	0.01853	0.0179
0.1	0.00041	0.00049	0.00731	0.01294	0.01043	0.01987	0.0134
0.2	0.00028	0.0003	0.00639	0.00779	0.00697	0.02055	0.0156
0.5	0.00021	0.00024	0.00524	0.00716	0.00576	0.02081	0.0152
1.0	0.0003	0.00039	0.00611	0.00805	0.00593	0.02392	0.0159
100.0	0.00025	0.00024	0.00618	0.00884	0.00675	0.02563	0.0230

* SKD-test from [37]
