# Supplementary material for: Coalescent Tree Imbalance and a Simple Test for Selective Sweeps Based on Microsatellite Variation
Source: PLoS Comput Biol. 2013 May 16;9(5):e1003060. doi: 10.1371/journal.pcbi.1003060 (PMC3656098; doi:10.1371/journal.pcbi.1003060)
Supplement: Table S12 — Empirical false positive rate. Population sub-structure with two sub-populations, split time in the past and sampling scheme , . Varying migration rate per generation per individuals. Significance levels are based on theoretical formulae according to eqs (7) and (8). (PDF) [file pcbi.1003060.s016.pdf]

Table S12. Empirical false positive rate. Population sub-structure with two sub-populations, split time $t = 1$ in the past and sampling scheme $n_1 = 150$, $n_2 = 50$. Varying migration rate m per generation per $4N$ individuals. Significance levels α are based on theoretical formulae according to eqs (7) and (8).

	$\alpha = 0.01$			$\alpha = 0.05$			
m	$T_2^{(\mathrm{sum})}$	$T_2^{(\mathrm{product})}$	$T_0^{(\mathrm{dist})}$	$T_2^{(\mathrm{sum})}$	$T_2^{(\mathrm{product})}$	$T_0^{(\mathrm{dist})}$	SKD*
0.0010	0.00037	0.00034	0.00646	0.00971	0.00696	0.02802	0.0960
0.0020	0.00034	0.00034	0.00648	0.00955	0.00696	0.02831	0.1007
0.0030	0.00038	0.00033	0.00684	0.00922	0.00691	0.02932	0.1014
0.0040	0.0004	0.00042	0.00689	0.01001	0.00745	0.02877	0.1060
0.0050	0.00036	0.00031	0.00644	0.00942	0.00667	0.02811	0.1008
0.0060	0.00038	0.00028	0.00613	0.00972	0.00699	0.02753	0.0940
0.0070	0.00028	0.00033	0.0063	0.00964	0.00686	0.02915	0.0975
0.0080	0.00043	0.00042	0.00687	0.00957	0.00736	0.02924	0.1052
0.0090	0.00031	0.00029	0.00669	0.00995	0.00747	0.02911	0.1029
0.01	0.00036	0.00033	0.00677	0.00919	0.00707	0.02839	0.0996
0.02	0.00035	0.00038	0.00664	0.00935	0.00699	0.02837	0.1029
0.03	0.00031	0.00036	0.00654	0.0095	0.00701	0.02894	0.1021
0.04	0.00037	0.00037	0.00644	0.01004	0.00743	0.02807	0.1063
0.05	0.00031	0.00032	0.007	0.00953	0.00694	0.02816	0.0976
0.06	0.00041	0.00038	0.00701	0.00982	0.00719	0.02869	0.1018
0.07	0.00025	0.00021	0.00629	0.00989	0.007	0.02872	0.0990
0.08	0.00043	0.00044	0.00688	0.00977	0.00733	0.02924	0.1043
0.09	0.00034	0.00033	0.00694	0.00973	0.00744	0.02865	0.0990
0.1	0.00033	0.00031	0.0067	0.00934	0.00715	0.02896	0.0972
0.2	0.00027	0.00033	0.00721	0.00951	0.00681	0.02946	0.0972
0.3	0.00021	0.00031	0.00663	0.0086	0.00643	0.0286	0.0913
0.4	0.00033	0.00038	0.00668	0.00896	0.00699	0.02856	0.0917
0.5	0.00033	0.00042	0.00686	0.00953	0.00689	0.02883	0.0911
0.6	0.00032	0.00035	0.0071	0.00885	0.00654	0.02862	0.0874
0.7	0.00032	0.00036	0.00672	0.00828	0.00659	0.02913	0.0838
0.8	0.00045	0.0004	0.00646	0.00846	0.00689	0.0277	0.0872
0.9	0.00038	0.00045	0.00668	0.00871	0.00716	0.02889	0.0832
1.0	0.00034	0.00041	0.00639	0.00881	0.00694	0.02835	0.0774
1.5	0.00038	0.00021	0.00668	0.00797	0.00635	0.02848	0.0818
2.0	0.00029	0.00034	0.0066	0.00812	0.00644	0.02843	0.0692
4.0	0.00029	0.00031	0.00641	0.00738	0.00611	0.02779	0.0650
6.0	0.00033	0.00027	0.00629	0.00745	0.00647	0.02716	0.0583
8.0	0.00026	0.00022	0.0068	0.00671	0.00575	0.02824	0.0536
10.0	0.00022	0.0003	0.00633	0.00666	0.00537	0.02645	0.0514

* SKD-test from [37]
