# Supplementary material for: Coalescent Tree Imbalance and a Simple Test for Selective Sweeps Based on Microsatellite Variation
Source: PLoS Comput Biol. 2013 May 16;9(5):e1003060. doi: 10.1371/journal.pcbi.1003060 (PMC3656098; doi:10.1371/journal.pcbi.1003060)
Supplement: Table S14 — Empirical false positive rate. Mutation model with jumps of size . Varying probability for a step of size . With probability the step size is . Significance levels are based on theoretical formulae according to eqs (7) and (8). (PDF) [file pcbi.1003060.s018.pdf]

Table S14. Empirical false positive rate. Mutation model with jumps of size 7. Varying probability p for a step of size 7. With probability $1-p$ the step size is 1. Significance levels α are based on theoretical formulae according to eqs (7) and (8).

	$\alpha = 0.01$			$\alpha = 0.05$			
p	$T_2^{(\text{sum})}$	$T_2^{(\text{product})}$	$T_0^{(\text{dist})}$	$T_2^{(\text{sum})}$	$T_2^{(\text{product})}$	$T_0^{(\text{dist})}$	SKD*
0.0010	0.0006	0.00056	0.00904	0.01013	0.00917	0.03397	0.05994
0.0020	0.00064	0.00079	0.0124	0.01245	0.01149	0.04063	0.06283
0.0050	0.00117	0.00156	0.01743	0.01532	0.01705	0.05315	0.07643
0.01	0.00141	0.00213	0.02254	0.02011	0.0233	0.06715	0.09604
0.02	0.00226	0.00327	0.02432	0.02711	0.03064	0.07248	0.13298
0.05	0.00252	0.00315	0.01724	0.02917	0.03067	0.06025	0.20621
0.1	0.00191	0.00199	0.01178	0.02452	0.02395	0.04676	0.26965
0.2	0.00129	0.00107	0.00825	0.01841	0.01529	0.03595	0.30976
0.5	0.00062	0.00047	0.00661	0.01194	0.00915	0.03049	0.34659
1.0	0.00045	0.00029	0.00658	0.00886	0.00715	0.02886	0.05459

* SKD-test from [37]
